# Mapping Behavioral Health Serious Game Interventions for Adults With Chronic Illness: Scoping Review

**DOI:** 10.2196/18687

**Published:** 2020-07-30

**Authors:** Teresa Hagan Thomas, Varshini Sivakumar, Dmitriy Babichenko, Victoria L B Grieve, Mary Lou Klem

**Affiliations:** 1 School of Nursing University of Pittsburgh Pittsburgh, PA United States; 2 School of Computing and Information University of Pittsburgh Pittsburgh, PA United States; 3 Department of Pharmacy and Therapeutics School of Pharmacy University of Pittsburgh Pittsburgh, PA United States; 4 Health Sciences Library System University of Pittsburgh Pittsburgh, PA United States

**Keywords:** review, chronic disease, behavioral sciences, video games

## Abstract

**Background:**

Serious games for health are increasingly being used to address health outcomes in patients with chronic illnesses. These studies vary in their study designs, patient populations, frameworks, outcome variables, and degree of specificity of the serious game intervention.

**Objective:**

This scoping review aims to clarify the conceptual features of the existing research related to serious games designed to improve cognitive and behavioral outcomes in adults with chronic illness.

**Methods:**

We applied the Preferred Reporting Items of Systematic Reviews and Meta-Analysis for scoping reviews (PRISMA-ScR) methodology, including an a priori research question. We searched 4 electronic databases to identify articles published through November 2019. Inclusion criteria encompassed (1) adults 18 years or older; (2) patients with a diagnosis of chronic illness; (3) a serious game intervention; and (4) defined patient outcomes that assess patients’ behavioral, cognitive, or health outcomes.

**Results:**

Of the 3305 articles identified, 38 were included in the review. We charted and analyzed the theoretical frameworks, key concepts, and outcome variables of these studies with summaries of features across articles. The majority of studies used a randomized controlled trial design (23/38, 61%), included a custom serious game intervention (22/38, 58%), and lacked a theoretical framework (25/38, 66%). Common outcome variables included quality of life (16/38, 42%), mood (15/38, 39%), cognitive function (13/38, 34%), symptoms (12/38, 32%), and physical activity (9/38, 24%). Key differences between studies included whether or not serious games aimed to train versus teach patients, be widely accessible versus tailored interventions, or replace versus complement current treatments.

**Conclusions:**

This scoping review defines the current landscape of research in serious games for health research targeting behavioral and cognitive outcomes in adults with chronic disease. Studies have addressed a variety of patient populations and diverse patient outcomes. Researchers wanting to build on the current research should integrate theoretical frameworks into the design of the intervention and trial to more clearly articulate the active ingredients and mechanisms of serious games.

## Introduction

### Background

Over the past decade, health-oriented clinical and research apps using electronic serious games have increased as a means to improve patient outcomes and provide health education [1]. Serious games take important health topics traditionally taught to patients and apply game features to provide a motivational, engaging, and even fun learning experience [2]. These games may be used to prevent disease [3], improve the health of patients with disease [4], and enhance social interaction to improve health [5]. Additional genres of serious games teach medical professionals skills [6-8], ultimately attempting to improve patient outcomes by improving clinician knowledge. A central attraction of electronic serious games is their ability to educate, motivate, and involve users without using conventional patient education that relies heavily on less engaging and often more intense training, such as written instruction or one-on-one consultation. The potential benefits of serious games include their high level of learning engagement, ease, and low cost of dissemination and distribution [9].

McGonigal [10], a game designer and leader in developing games to improve quality of life, defined serious games as having 4 hallmark features: (1) an *overall* *goal—*some desired outcome that provides a sense of purpose, (2) *rules—*limitations on the users’ activities that necessitate creativity and strategic thinking, (3) *feedback system—*a way to communicate with users about their proximity to achieving a goal that motivates and promises that the goal is achievable, and (4) *voluntary participation—*the freedom to enter and leave the game so that participation is safe and pleasurable. The theorized learning mechanism leading serious games to be an effective teaching tool involves immersive qualities in which the users becomes engrossed in the game; the requirement for users to learn skills in increasingly difficult challenges; and use of the user’s desire for mastery, arousal, diversion, and challenge [10,11].

Transformational games have emerged as a subset of serious games that try to positively impact the user by addressing outcomes, including behaviors, attitudes, and social issues [12]. Unlike traditional educational games that focus on the game as an end in itself, transformational games aim to have users learn through intentional participation in narratives that employ concepts that, if successfully learned, should extend beyond the game and meaningfully impact their lives [13]. This generalization requires that the game have specific behavior and learning outcomes, including an explicit plan for how users will transfer their skills from the game into real-world settings.

Rehabilitation and behavioral sciences have pioneered novel motivational, engaging games. Currently, the field is rapidly expanding to new patient populations and topics. Patients with chronic illnesses such as cancer, cardiovascular disease, diabetes, and obesity face long-term health problems that require diligent management. For these patients, behavioral, cognitive, and health outcomes are essential in ensuring that they can self-manage their illness and prevent long-term morbidity and mortality. These patient outcomes cut across different chronic diseases and include physical activity, maintenance of a healthy body weight, quality of life, symptom burden, mood, and cognitive function.

As electronic serious game interventions for health have increased, a lack of attention to the theoretical underpinnings has resulted in disparate game mechanisms, unclear theoretical frameworks of action, and weak study designs. Previous review articles have noted a need for improved research methods and expanded clinical applicability [14-17]. A fresh review of serious games in adults with chronic illnesses can assist researchers and clinicians in recognizing the strengths and limitations of how these studies have been designed. AA review can also identify ways to improve research rigor and translation to improved patient outcomes. Mapping the current research landscape will assist in establishing the key concepts, variables, theories, and frameworks undergirding this growing body of research.

### Objective

Scoping reviews allow investigators to systematically examine emerging areas of evidence and can help identify gaps in knowledge, clarify concepts, and reveal methodological concerns in new areas of research. As scoping reviews generally have broader inclusion criteria than systematic reviews without an assessment of study quality, they allow for findings from disparate patient populations and contexts and a more comprehensive determination of evidence. Researchers and clinicians can apply the findings of a scoping review to more astutely build on the current evidence base and address gaps in existing research. Although systematic reviews of serious games have been conducted within specific patient populations and health care settings [18-21], the evidence supporting serious games focused on health skills and behaviors among adults with chronic diseases have not been mapped systematically. This scoping review aimed to define the concepts applied to studies using serious games to improve the health of adults with chronic illnesses.

## Methods

### Research Question

Our *a priori * research question was as follows: “What types of theoretical frameworks, key concepts, and outcome variables exist within serious game interventions to improve the cognitive and behavioral outcomes of adult patients with chronic illness?”

### Protocol

We followed a scoping review methodology to synthesize concepts and research concerning the use of serious games as interventions designed to address cognitive and behavioral outcomes among adult patients with chronic illness. The objectives, inclusion criteria, and methods for this scoping review were specified in advance and documented in a protocol. Our protocol is freely available through the Open Science Framework [22]. This protocol follows the Preferred Reporting Items of Systematic Reviews and Meta-Analysis for scoping reviews (PRISMA-ScR) methodology to ensure that our results would be systematically conducted with minimal bias [23].

### Inclusion Criteria

To be included in the scoping review, articles needed to focus on the following: (1) adults 18 years or older; (2) patients with a diagnosis of chronic illness; (3) a serious game intervention; and (4) defined patient outcomes that assess patients’ behavioral, cognitive, and health outcomes.

### Participants

We used definitions of chronic illness as defined by the Centers for Disease Control and Prevention [24] or the Center for Medicare and Medicaid Services [25]. These illnesses include Alzheimer's disease, arthritis, cancer, cardiovascular diseases including hypertension and stroke, chronic lung disease including asthma and chronic obstructive pulmonary disease (COPD), cystic fibrosis, diabetes, epilepsy and seizures, obesity, and oral health. Some articles included participants with illnesses that require long-term maintenance and ongoing medical care but were not listed in these definitions [26]. To err on the side of inclusion, we revised our definition of chronic illness to include additional diseases that could be considered chronic, such as Parkinson’s disease. When studies included participants with and without chronic illnesses, we reviewed the full-text article to determine the proportion of participants with chronic illnesses and only included articles in which the majority of participants had an eligible disease.

### Concept

We broadly defined serious games as “games that are designed to entertain players as they educate, train, or change behavior” [27]. We used the 4 criteria by McGonigal [10] listed above to determine whether an intervention included the requisite elements of a serious game. We did not stipulate that the serious game required any specified dose or intensity, length, or use of a comparison group. When limited descriptions of the game were available, we made assumptions about the existence of certain features to be liberal in article selection. Some articles included screenshots of the serious game, which indicated that the game included feedback, points, and engaging characters and scenery despite the article not describing these features. We did not include articles for which the serious game was not the intervention but was conceptualized as a diagnostic tool or priming event for a separate intervention. For example, some studies used video games to prime study patients' working memory and cognitive abilities, but the study was not designed to test the effect of that video game on patients’ outcomes.

### Outcomes

We included any study that assessed patients’ behavioral, cognitive, and health outcomes. We created an ongoing list of outcome variables based on the variables identified in specific studies. Examples of behavioral outcomes included physical activity, medication adherence, and self-management.Examples of cognitive outcomes included executive function, working memory, learning new knowledge, and motivation. Examples of health outcomes included quality of life, mood, symptoms, BMI, hemoglobin A_1c_ (HbA_1c_), and blood pressure. We did not include functional outcomes (eg, the impact of a serious game on hand function or range of motion) because these assessments did not include the patient’s involvement in learning or behaving to manage their chronic disease.

### Context

As many interventions and meta-analyses have studied the impact of games to increase patients’ mobility and functional outcomes [28] and do not include behavioral or cognitive outcomes, we only included serious game interventions that included behavioral, cognitive, or related health outcomes. For example, a study that analyzed the effect of a serious game on patients’ motor control would not be included if the main outcomes only included functional measurements. A study would be included if the main outcomes included measures of patient self-management of their illness or symptoms.

### Identifying Relevant Studies and Study Selection

The following databases were searched: PubMed (1946 to November 28, 2017), EbscoHost CINAHL (Cumulative Index of Nursing and Allied Health Literature; 1981 to November 2017), Ovid PsycINFO (1967 to November, Week 2, 2017), and EMBASE (Excerpta Medica dataBASE; 1974 to November 28, 2017). All database searches were run on November 28, 2017, and, when available, a search limit to the English language was applied. Updated searches were run on November 25, 2019. An experienced health sciences librarian (MK) designed the PubMed search [22] and then translated that search for use in the other databases. The search strings consisted of natural language terms and (when available) controlled vocabulary representing the concepts of “videogames,” “serious games,” and “chronic diseases.”

Two individuals independently screened abstracts and full-text articles using DistillerSR software (DistillerSR, Evidence Partners). This software allowed reviewers to collaboratively review all abstracts for the inclusion criteria. For all included articles, 2 reviewers extracted data and agreed upon the results. When reviewers disagreed on the inclusion criteria or data, they met to discuss the articles until consensus was reached.

Articles that met the inclusion criteria were charted to provide a descriptive summary. We extracted data including their study design; patient population; frameworks; behavioral, cognitive, or health-related outcomes; and a description of the serious game. We further described the key concepts that emerged as similarities and differences across the studies.

## Results

**Figure 1:**
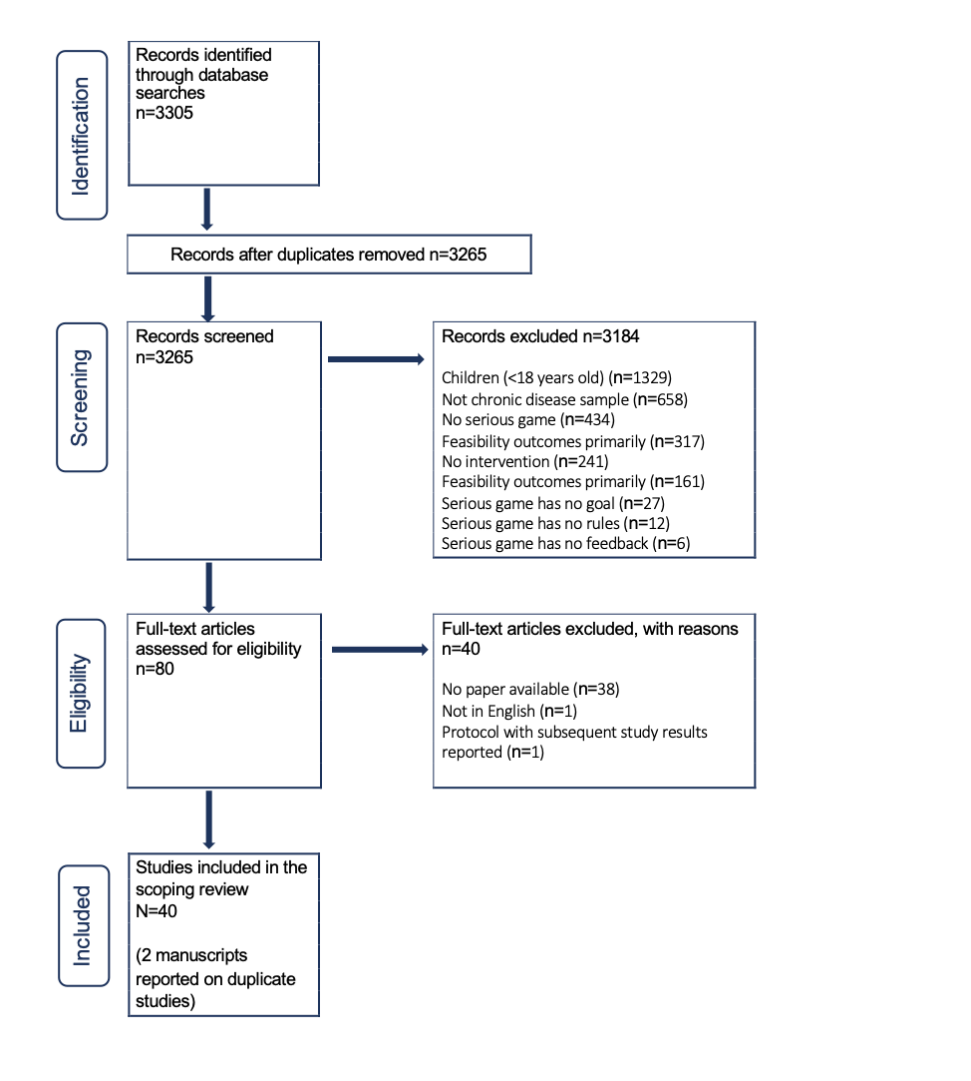
Preferred Reporting Items of Systematic Reviews and Meta-Analysis for scoping reviews flowchart.

The search strategy yielded 3268 references, of which 40 met the inclusion criteria. Figure 1 illustrates the PRISMA-ScR flowchart for our results. One research study had 2 manuscripts reporting different results from the same trial. As the framework, sample, and intervention were the same despite reporting on different outcome variables, we grouped these manuscripts as one study. A separate group of investigators had 2 manuscripts for the same trial and a separate manuscript reporting the study protocol. We grouped these manuscripts together, prioritizing the trial results. Our final sample included 38 studies. Table 1 illustrates the frequency of trial designs, populations, and most frequently cited study outcomes.

**Table 1:** Frequency of trial designs, patient populations, and study outcome variables.

Characteristics		Values
**Trial design, n (%)**		
	Randomized controlled trial	23 (61)
	Feasibility study	8 (21)
	Protocol for study	6 (16)
	Case study	1 (3)
**Patient population, n (%)**		
	Stroke	17 (45)
	Cancer	6 (16)
	Diabetes	5 (13)
	Dementia	3 (8)
	Hypertension	3 (8)
	Parkinson’s disease	2 (5)
	Obesity	1 (3)
	Chronic obstructive pulmonary disease	1 (3)
**Outcome variable, n (%)**		
	Quality of life	16 (42)
	Mood	15 (39)
	Cognitive function	13 (34)
	Symptoms	12 (32)
	Physical activity	9 (24)

### Trial Designs

The majority of studies (23/38, 61%) reported the results of randomized controlled trials, whereas 16% (6/38) articles were protocols for clinical trials. Of these articles, the comparison groups for these trials mostly included a conventional form of active therapy (23/29, 79%), such as rehabilitation, rather than usual care or attention control (6/29, 21%). Few studies in the stroke population used a usual care comparison group, opting for conventional rehabilitation as a comparison. Less frequent trial designs included one-group pretest/posttest feasibility evaluations (8/38, 21%) and a case study (1/38, 3%).

### Populations

The patient populations represented a diverse group of common chronic diseases, including stroke (17/38, 45%), cancer (6/38, 16%), diabetes (5/38, 13%), dementia (3/38, 8%), hypertension (3/38, 8%), Parkinson’s disease (2/38, 5%), obesity (1/38, 3%), and COPD (1/38, 3%). The mean sample size was 82 patients (SD 104), with a range of 1 to 456. Most studies had small samples that were not adequately powered to test for statistically significant differences.

### Serious Games

The majority of studies (22/38, 58%) described serious games that were custom-made interventions for specific patient populations. Studies describing custom-made games tended to have an in-depth description of the game’s features, often because the research team built the game before evaluating it. Three custom-made games used a version of the BrightArm virtual reality system [29-31]; all other games were evaluated in separate manuscripts.

The remaining studies (16/38, 42%) used off-the-shelf games designed for the general population. Off-the-shelf games included games within the Nintendo Wii Fit (11/38, 28%), Xbox Kinect (3/38, 8%), and other off-the-shelf software (2/38, 5%). Often, the authors did not explicitly state the features of the off-the-shelf games. In lieu of such detail, we assumed that games such as bowling, tennis, and ping pong had a goal (score the most points), restrictions on actions (only specific actions are allowed), and feedback (points, badges, and scoreboards). Although these off-the-shelf games are commonly used as exercise, physical activity, and mobility interventions, different studies specified different rationales for the same technology. For example, different studies used the Nintendo Wii Fit game suite to improve patient motor function, balance, cognition, or general physical activity. Of the 17 studies reporting on patients recovering from a stroke, 8 (47%) used off-the-shelf games.

### Theoretical Frameworks

Most of the articles (25/38, 66%) lacked a clear theoretical framework. This was especially true for articles focused on patients with a history of stroke; only 3 of the 17 studies included a theoretical framework [31-33]. In most cases, the behavioral or cognitive outcomes appeared to serve as an *add-on* to other functional outcomes without an underlying hypothesis proposing how the serious game leads to improved outcomes. For example, authors may include measures of quality of life, depression, or motivation to their study but not indicate how these health outcomes related to the intervention.

Within the 34% (13/38) studies that cited a theoretical framework, frameworks varied in their application to the intervention and variables. Most frameworks focused on mechanics of specific interventions (simulation theory [33], cognitive training reorganization on brain network infrastructure [34], spaced education [35], neurofeedback for pain control [36], integrative rehabilitation [31], and self-management [33,37]). Other frameworks focused on intervention development (intervention mapping approach [38] and Gagne’s instruction strategies [39]) and rationales for serious game features (behavioral economics [40,41], dual-task training [32], narrative transportation theory [42], self-determination theory [42-44], and behavior change theory [43,44]).

### Outcome Variables

Studies varied greatly in the type and number of outcome variables used to assess the efficacy of the serious game. We report the most frequently cited outcome variables while noting several additional outcome variables. Multimedia Appendix 1 [29-68] includes a complete list of the behavioral, cognitive, and health outcomes as conceptualized within each study. We indicate whether the data for each outcome are based on patient self-report; examination with a valid assessment or clinical assessment; and/or objectively collected through sensor data, blood work, or medical records.

#### Quality of Life

Quality of life (including health-related quality of life) was the most common outcome across all studies (16/38; 42%), including a variety of chronic diseases. Many studies included quality of life as an outcome, seeming to want to capture a patient’s overall well-being. The rationale for including quality of life was rarely described. It was frequently captured as a secondary outcome to functional outcomes of mobility and strength in rehabilitation serious games for patients recovering from a stroke. Quality of life measures included self-report surveys, including the EuroQol-5 Dimension, Short Form Survey-36, and Stroke Impact Scale.

#### Mood

Mood—including depression and anxiety—was included as an outcome in 39% (15/38) studies. Similar to quality of life, mood variables were often included with minimal description tying these outcomes to the use and intent of the serious game intervention. Studies frequently included the Beck Depression Inventory as an outcome of interest, although the rationale for including this scale was lacking, and therefore, the discussion of these findings was minimal. Other studies measured patients’ self-reported anxiety using the State-Trait Anxiety Inventory or disease-specific mood surveys.

#### Cognitive Function

Many serious games have been designed to improve memory and attention in patients with a history of stroke, dementia, and Parkinson’s disease. Cognitive function was a common outcome in studies (13/38, 34%), especially for serious games targeting patients with dementia and stroke. Cognitive function assessments commonly included the Trail Making Test, Neuropsychological Assessment Battery, Mini-Mental Status Examination, and other neuropsychological evaluations that were administered by a trained study team member.

#### Symptoms

Physical symptoms such as fatigue and pain were reported as outcomes of the serious game in 32% (12/38) studies. Specific symptoms varied widely and were typically tailored to the patient population within the article (eg, fatigue was commonly assessed for patients with cancer [38,42,49,50]). Symptom measures included the Functional Assessment of Cancer Therapy, McGill Pain Questionnaire, and other self-report measures. Some studies measured symptoms using patient medical records that indicated symptom ratings and medication use to manage symptoms.

#### Physical Activity

A total of 24% studies (9/38) included a measure of physical activity as an outcome variable. In this case, physical activity was considered an activity of diabetes self-management, which would lead to better control of diabetes. Physical activity measures ranged from passive sensor data from wearable devices (eg, pedometers, accelerometers, and sensors on phones), clinical examinations of patients’ cardiorespiratory fitness, to self-reported activity surveys.

#### Other Outcomes

Studies assessed myriad additional outcomes. Several studies assessed patients’ motivation (5/38, 13%) in engaging in a serious game, captured by various self-report measures. Several studies assessed physical measurements conceptualized as health outcomes of desired behaviors. For example, studies targeting patients with diabetes often collected blood levels of HbA_1c_ (4/38, 11%) as an outcome variable indicating whether or not patients improved their average blood sugar level. Similarly, 3% study (1/38) assessed blood pressure as a target health outcome for patients with hypertension. Additional outcomes included patient self-reports of education or knowledge, self-management, medication adherence, and other outcomes. A minority of studies included outcomes related to learning within a serious game, fidelity of the intervention, or transference of learning within the game to real-life settings.

### Key Concepts

#### Teaching Versus Training

A significant difference existed between the serious games that were described as teaching patients a specific behavior or skill compared with games intended to train patients in movements or activities. Games that were focused on teaching tended to have more explicit descriptions tying the features of the serious game to the intended learning outcomes, whereas games that were focused on training tended to have minimal explanation of how the game would train patients. Several studies employing a teaching approach cited theoretical frameworks linking how learning a behavior would lead to improved outcomes. For example, Kerfoot et al [35] explicitly stated how a spaced education and self-management framework would teach patients how to manage their diabetes, thereby reducing their HbA_1c_ and diabetes distress and increasing their knowledge of diabetes.

#### Accessibility Versus Tailored

Differences between the off-the-shelf and custom-made serious games was a consistent concept discussed across the studies. Many studies cited the advantages of using an off-the-shelf serious game such as Nintendo Wii Fit or Xbox Kinect, including easy integration into community settings and dissemination of effective protocols. Alternatively, studies in which the serious game was tailored to a specific patient population or health problem discussed the advantages of integrating specific content and mechanisms known to be important and relevant to that patient population. Tailoring may include specific technologies required to address specific health problems or a more general consideration of the psychosocial needs of the population. The Memory Matters game by Yu et al [68] intricately designed their interactive reminiscence game for patients with dementia, providing objects, images, and music familiar to the target patient population.

#### Replacement Versus Complementary

Several studies described the rationale for serious game intervention as a replacement for conventional treatment. In many cases, serious games were seen as a substitute for occupational or rehabilitation therapy in patients recovering from a stroke. Shin et al [61] created the RAPAEL Smart Glove to simulate upper extremity rehabilitation and compared it with conventional occupational therapy. This varied vastly from serious games meant to provide additional, novel, and meaningful support but otherwise not replacing an existing intervention, such as interventions focused on teaching self-management or coping skills. For example, Höchsmann et al [43,44] reported on their intervention—MOBIGAME—which aimed to reduce diabetes by engaging patients in an immersive, relaxing program focused on increasing physical activity, motivation, and adherence.

## Discussion

### Primary Findings

This scoping review identified that electronic serious games for patients with chronic illness target a variety of patient populations, are mostly custom-made games, largely lack theoretical frameworks, and measure a broad array of patient outcomes. Common themes across studies included whether or not games were intended for teaching versus training purposes, meant to be widely available versus tailored to patient populations, and replace or complement existing therapies.

The 38 studies in this review represented 8 different patient populations, indicating that serious game interventions are applicable across diverse chronic illnesses. Many of the studies were designed for patients recovering from a stroke, possibly because off-the-shelf games (used in almost half of the studies for patients with stroke) included physical and functional targets similar to those used in conventional rehabilitation therapy. Nonetheless, a variety of serious games have been used across and within populations with chronic illness, underscoring the diversity of designs and settings in which researchers are investigating the efficacy of serious games.

A major limitation of the current landscape of serious games for adults with chronic illness is the overwhelming lack of theoretical frameworks. Two-thirds of studies did not cite a theoretical framework guiding the intervention, trial design, or proposed mechanisms linking the serious game to patient outcomes. This finding is similar to a recent systematic review of serious games [69]. One implication of this lack of frameworks is the broad list of study outcomes and lack of mechanistic or fidelity assessments. Outcomes were measured using diverse self-report measures, examinations, and sensor data, limiting the potential to compare outcomes across studies. The most frequently included outcomes, including quality of life and mood, were added without explicit hypotheses or proposed mechanisms linking them to the serious game. This finding corroborates other research [70], including a scoping review by Rohrbach et al [71], which found that virtual reality interventions for patients with stroke often include affective traits such as motivation and enjoyment without integration into the overall design and mechanistic planning of the intervention.

Recent consensus statements for the consistent integration of theoretical frameworks into serious games for health research provide researchers, developers, and clinicians with explicit recommendations on how to address this limitation of current studies [72]. Ideally, serious games for health would leverage the active ingredients of games (immersive, challenging, and chance for mastery) with persuasive strategies commonly used in behavior change research. Reassuringly, 5 of the 9 (56%) studies published in 2019 cited a theoretical framework, perhaps indicating a trend in serious game research. In the future, research should integrate gamification and behavior change theory into rigorously designed trials based on theoretical frameworks [73].

Few studies have assessed the transfer of skills learned within a serious game into real-life settings. Although some studies assessed patient behaviors to determine whether the serious game led to changes in lifestyle and activity, this was infrequent and sometimes only assessed during the intervention period. A systematic review by Kuipers et al [74] noted a lack of attention to the mechanistic underpinnings of how serious games facilitate learning and drive their intended long-term effects. The authors urge researchers to consider game transference effects in the design of serious games for health, increasing the likelihood—or at least the ability to document—that these health interventions will lead to lasting desired patient behaviors and outcomes. Only one study addressed how a serious game was integrated into patient care via an existing research and clinical platform [40]. Researchers designing custom serious games for health should include upfront plans for disseminating their intervention within clinical care or broadly into larger populations.

The outcome variables of the studies included were inconsistently aligned with the serious game’s goals. For example, games designed to improve cognition and physical fitness measured outcomes of executive function and physical activity, respectively. On the other hand, many studies included outcomes that were less clearly aligned with the intervention. This was especially true for studies that measured quality of life and mood. Many of these studies did not indicate a rationale for why quality of life or mood would change because of the serious game but appeared to be included as measures of patients’ general well-being.

This scoping review has limitations. First, this review relied on published manuscripts of electronic serious games. We attempted to include a broad definition of both electronic serious games and chronic illnesses, although fluctuations in the terminology around these terms limited our search strategies’ abilities to identify articles. Conference proceedings did not include sufficient detail to answer our research questions. Analog serious games were not included in this review and could be considered in future analyses. Second, this review relied on descriptions of serious games and interventions described within manuscripts, which were often limited, especially concerning game features and frameworks. Although we attempted to use our best judgment, additional details of the games and interventions could provide a more comprehensive summary. Finally, the broad nature of this review—although appropriate for a scoping review—limits the ability to provide specific conclusions based on patient population, game design, patient outcomes, etc. Future research could include more broader definitions of serious games, request information from authors to clarify game features and frameworks, and assess the efficacy of the serious game.

### Conclusions

This review assists researchers in creating serious game interventions to address chronic health conditions by providing clarity on how to build from the current structure of serious games for health research. Researchers should continue the existing momentum in building robust, large trials driven by theoretical underpinnings of how interventions are hypothesized to impact outcomes. As attention to how game features lead to behavioral, cognitive, and health outcomes increases, we as a field will grow in the ability of our research to effectively and efficiently impact the most significant health problems patients experience.

## Appendix

Multimedia Appendix 1 Results table of studies of serious games for adults with chronic disease targeting behavioral, cognitive, or other health outcomes (N=38).

## Abbreviations

COPD: chronic obstructive pulmonary disease
HbA_1c_: hemoglobin A_1c_
