# Supplementary material for: Mapping Behavioral Health Serious Game Interventions for Adults With Chronic Illness: Scoping Review
Source: JMIR Serious Games. 2020 Jul 30;8(3):e18687. doi: 10.2196/18687 (PMC7426803; doi:10.2196/18687)
Supplement: Multimedia Appendix 1 [file games_v8i3e18687_app1.docx]

**Multimedia Appendix 1.** Results table of studies of serious games for adults with chronic disease targeting behavioral, cognitive, or other health outcomes (N=38).

| Reference | Study purpose | Study population, sample | | Study design | Framework | Serious game and purpose within study | Serious game description and features (goal, mechanics, feedback, and mood) | Cognitive, behavioral, and health outcomes |
| --- | --- | --- | --- | --- | --- | --- | --- | --- |
| Adie et al [28] | “This article reports the efficacy of using the Wii™ to improve affected arm function for patients after stroke in a home setting in the United Kingdom” | Stroke, N=235 | | Clinical trial with randomization into 2 groups: serious game or tailored arm exercises | None | Nintendo Wii Fit, training that is repetitive and motivational as an adjunct to stroke rehabilitation | Game features assumed | Quality of life (multiple self-report surveys) |
| Adomavičienė et al [29] | “The objectives of the study are to clarify in which area of functional upper limb recovery these new technologies are more suitable and effective and how much these interventions affect functional state and cognitive functions” | Stroke, N=42 | | Clinical trial with randomization into 2 groups: virtual reality–based Kinect-based system or conventional therapy with Armeo spring robot-assisted trainer | None | Microsoft Kinect, movement execution | Game features assumed | Cognitive function (examination), mood (self-report) |
| Burdea et al [30] | “To describe the development of BrightBrainer™ integrative cognitive rehabilitation system and determine clinical feasibility with nursing home-bound dementia patients” | Dementia, N=10 | | One-group, pretest and posttest feasibility study | None | Bright Arm rehabilitation table, assessment and cognitive rehabilitation of individuals with mild cognitive impairment or with early stage Alzheimer’s disease | - Mechanics: 3 attention games, 2 games to train memory, and 1 game for decision-making. - Action points: hitting ball with paddle, hitting mallets, etc. - Mood: music. - Feedback: summative scores | Cognitive function (examination), mood (self-report) |
| Choi et al [31] | “The aim of this study was to determine whether commercial gaming-based virtual reality therapy was as effective as conventional Occupational Therapy for functional recovery of the hemiparetic upper extremity and attention deficits in subacute stroke patients” | Stroke, N=20 | | Clinical trial with randomization into 2 groups: serious game or occupational therapy | None | Nintendo Wii Fit, motor recovery including cognitive training | - Mechanics: completing games including swordplay, table tennis, and canoe games | Cognitive function (multiple examinations) |
| Choi et al [32] | “The objective of the present study was to develop a mobile game-based upper extremity virtual reality program for patients who have experienced stroke, and to evaluate the feasibility and effectiveness of the program” | Stroke, N=24 | | Clinical trial with randomization into 2 groups: serious game intervention or intervention plus conventional occupation therapy | None | MoU-Rehab: was designed to improve strength, endurance, range of motion, control, speed, and accuracy of movement in the upper extremity | - Mechanics: 4 games tailored based on function. - Feedback: visual and auditory | Quality of life (self-report), mood (self-report) |
| da Silva Alves et al [33,34]^a^ | “The objective of this study was to evaluate fatigue, strength, and median frequency of leg muscles in individuals with and without cancer who participated in an exergaming protocol using an Xbox360 Kinect” [33]  “This study aimed to compare the quality of life of people with cancer in different stages and the treatment of the disease with individuals without cancer, after the exergaming protocol using the Xbox 360 Kinect console” [34] | Cancer, mixed; N=45 | | Clinical trial with randomization into 3 groups based on health characteristics | None | Xbox Kinect, exergaming | - 3 games within Kinect used; intended to meet weekly exercise recommendations. - Game features assumed | Symptoms (fatigue; both self-report and examination), quality of life (self-report) |
| da Silva Ribeiro et al [35] | “The present study compared the effect of a rehabilitation treatment using the Nintendo Wii with conventional physical therapy to improve the sensorimotor function and quality of life of post-stroke hemiparetic patients” | Stroke, N=30 | | Clinical trial with randomization into 2 groups: serious game or conventional physiotherapy | None | Nintendo Wii Fit, balance | - Mechanics: tennis, hula-hoop, soccer, and boxing games. - Feedback: progress difficulty increases | Quality of life (self-report) |
| Fan et al [36] | “The purpose of this pilot study was to determine the effects of an affordable off-the-shelf virtual reality treatment based on the Wii on participants’ intrinsic motivation and their upper arm motor functions” | Stroke, N=20 | | Clinical trial with randomization into 3 groups: serious game, placebo board game, or no treatment | None | Nintendo Wii Fit, function | Game features assumed | Motivation (self-report), quality of life (self-report) |
| Fortunato et al [37] | “The objective of this article was to describe the protocol for the Influencing DIabetics to Adapt Behaviors related to Exercise and weighT by Enhancing Social incentives (iDiabetes) trial, which aimed to evaluate the effectiveness of gamification interventions that leverage insights from behavioral economics to enhance either supportive, competitive, or collaborative social incentives to improve glycemic control, promote weight loss, and increase physical activity among overweight and obese adults with type 2 diabetes” | Diabetes, N=361 | | Clinical trial with randomization into 4 groups: 1 control and 3 intervention (supportive vs competitive vs collaborative social incentives) | Behavioral economics | Way to Health, platform to increase daily step count, reduce weight, or decrease HbA_1c_^b^ (based on patient-selected goal) | - Mechanics: achieving weekly goals related to steps, weight, or HbA_1c_. - Mood: collaborative or competitive mood depending on randomization group.Feedback: point structure to gain or lose points based on completing tasks, leaderboards, emails with rankings, and social support person being emailed rankings | Physical activity (sensor data from wearable device), weight (wireless weight scale), HbA_1c_ (laboratory test) |
| Gamito et al [38] | “The current study assesses the effectiveness of a VR-based intervention for the cognitive training and rehabilitation of stroke patients following these different aspects of the rationale behind this form of intervention by comparing patients submitted to the intervention to patients in waiting list control” | Stroke, N=20 | | Clinical trial with randomization into 2 groups: serious game or wait-list control | None | Virtual reality of daily life activities to improve memory and attention | - Mechanics: completing daily life activities with increasing demands on memory and attention | Cognitive function (examination) |
| Gorini et al [39] | “Our primary aim is to evaluate the effect of neurofeedback on pain control in patients with lung cancer who have been recently operated on” | Cancer, N=80, planned | | Protocol for clinical trial randomized into 2 groups: serious game or usual care | Neurofeedback for relaxation training | MindWave, relaxation techniques, motivation neurofeedback | - Mechanics: games. - Mood: environment changes. - Action points: minigames build mental capacity to have focus and medication - Feedback: behavior ratings at the end of each session | Opioid consumption (medical record), pain intensity (self-report and x-ray), symptoms (pain and medical record), mood (self-report), infections (blood test) |
| Hickman et al [40] | “The aim of this article is to report the preliminary efficacy of a serious game for health to enhance blood pressure control among community-dwelling adults with hypertension” | Hypertension, N=144 | | Clinical trial with randomization into 2 groups: serious game or attention control using screen-based patient education | None | eSMART-HD provides cognitive and behavioral strategies to enhance communication with health care providers and improve chronic disease self-management | - Mechanics: interactions with avatar-based health care professionals and structured communication system. - Feedback: real-time suggestions tailored to patient | Blood pressure (examination) |
| Hӧchsmann et al [41,42]^c^ | “The aim of this randomized controlled trial is to assess the effect of the game on daily physical activity (steps/day) in physically inactive individuals with type 2 diabetes” [41]  “The purpose of this study was to investigate if the behavior change technique–based smartphone game can motivate inactive individuals with type 2 diabetes for regular use and thereby increase their intrinsic physical activity motivation” [42] | Diabetes, N=36 | | Protocol for randomized clinical trial.  Clinical trial with randomization into 2 groups: serious game or one-time lifestyle counseling | Self-determination, behavior change theory | MOBIGAME, designed for patients with or at risk for type II diabetes mellitus. The goal is to induce a healthier, more active lifestyle | - Mechanics: metaphor of restoring a garden used throughout game. - Strength, endurance, flexibility, and balance exercises. - Mood: relaxing garden. - Feedback: scores and rewards | Physical activity (sensor data from phone), cardiorespiratory fitness (examination), HbA_1c_ (examination) [41].  Motivation (self-report), physical activity (sensor data from phone) [42] |
| House et al [43] | “This study describes the BrightArm Duo virtual reality system and determines its clinical benefit for maintenance of upper extremity function in nursing home residents who are chronic poststroke” | Stroke, N=7 | | One-group, pretest and posttest feasibility study | None | Bright Arm therapy: rehabilitation system for stroke survivors | - Mechanics: 9 different games including a visual memory game, bouncing ball game. - Feedback: summative feedback at the end of each game; color changes. - Rewards: fireworks and congratulatory text or applause | Mood (self-report), cognitive function (examination) |
| House et al [44] | “This article presents the design characteristics of the BrightArm system, as well as its evaluation protocol and first feasibility study on a group of older adult stroke survivors” | Stroke, N=5 | | One-group, pretest and posttest feasibility study | Integrative rehabilitation | Bright Arm Duo, upper extremity rehabilitation | - Mechanics: 9 games with various goals. - Feedback: summative feedback at the end of each game; color changes. - Rewards: fireworks and congratulatory text or applause | Cognitive function (multiple examinations), mood (self-report) |
| Hung et al [45] | “The objective of this study was to compare the training and maintenance effects of 3 weight-shifting balance training programs (2 kinds of exergaming systems and 1 conventional weight-shifting training program) on cognitive function of subjects with chronic stroke” | Stroke, N=37 | | Clinical trial with randomization into 3 groups: Wii Fit, Tetrax biofeedback, or conventional weight shifting | None | Nintendo Wii Fit, maintenance of motor function | Five games within Wii Fit, game features assumed | Cognitive function (examination) |
| Kannan et al [46] | “The purpose of this study was to determine the efficacy of a six-week, high-intensity, tapered cognitive-motor exergame training paradigm compared with conventional balance training among community-dwelling persons with chronic stroke” | Stroke, N=24 | | Clinical trial with randomization into 2 groups: Wii Fit with cognitive tasks (cognitive-motor exergame training) or conventional balance training | Dual-task training paradigm (motor and cognitive tasks presented simultaneously) | Nintendo Wii Fit and cognitive tasks | - Physical: 5 games within Wii Fit, game features described (balance game, soccer, etc), including goals, rules, and feedback.Cognitive: games focused on semantic memory, word lists, mental arithmetic, etc | Cognitive function (examination) |
| Kempf et al [47] | “In a randomized controlled trial we investigated the hypothesis that autonomous use of the interactive exercise game Wii Fit Plus over a period of 12 weeks is able to improve HbA1c (primary outcome) as well as weight, cardiometabolic risk factors, physical activity and quality of life (secondary outcomes) in type II diabetes mellitus patients” | Diabetes, N=176 | | Clinical trial with randomization into 2 groups: serious game or usual care | None | Nintendo Wii Fit, physical activity | Game features assumed | Physical activity (self-report), quality of life (multiple self-report surveys), mood (self-report), HbA_1c_ (examination), weight (examination), cardiometabolic risk factors (examination) |
| Kerfoot et al [48] | “We hypothesized that a team-based spaced education game on diabetes self-management education topics delivered to patients with type 2 diabetes could generate sustained improvements in their HbA1c. To test this, we conducted a randomized trial with an active control group among veterans with diabetes living in the eastern U.S.” | Diabetes, N=456 | Clinical trial with randomization into 2 groups: serious game or serious game regarding civics content and paper diabetes self-management content | | Spaced education, self-management | Spaced education web-based game, case scenarios accompanied by multiple-choice questions | - Mechanics: respond to multiple-choice questions regarding scenarios related to diabetes self-managements - Action points: select answer. - Feedback: presentation of correct answer with explanation, individual points, team scores, and leaderboard | HbA_1c_^b^ (examination), empowerment (self-report), symptoms (distress, self-report), medication adherence (examination) |
| Kim et al [49] | “We aimed to investigate the effects of a serious game on depressive symptoms in breast cancer patients with depression using clinical scale measures and analyzing resting state networks” | Cancer, N=35 | Clinical trial with randomization into 2 groups: serious game or usual care | | None | Hit the cancer, reduce stress, mood encouragement, selective attention | - Mechanics: visualization of cancer cells tailored to patient and healthy cells. Differentiation between cancer and healthy cells becomes less clear in higher levels of game. - Action points: remove cancer cells and keep healthy cells | Mood (multiple self-report surveys), symptom (stress, self-report) |
| Kim et al [50] | “This study was a proof-of-concept randomized controlled trial aimed at evaluating the benefits of smartphone-based mobile game use in breast cancer patients receiving cytotoxic chemotherapy” | Cancer, N=76 | Clinical trial with randomization into 2 groups: serious game or conventional education | | Education regarding cancer symptom and self-management | ILOVEBREAST, mobile multiplayer, social network game to improve self-management and reduce the side effects of cancer treatment | - Mechanics: create avatar based on patient’s health history. Avatar completes quests including taking medication on time, cooking, and exercising. - Action points: taking medication on time. - Feedback: receive heart coins when quest is completed, with more coins meaning greater health; coins can be used to purchase items within the game | Medication adherence (self-report), symptoms (physical side effects, self-report), mood (multiple self-report surveys), quality of life (self-report) |
| Klompstra et al [51] | “The aim of this study was to introduce Nintendo Wii Sports to an older patient with heart failure at home and determine whether this influenced his daily physical activity, as well as to describe his experiences and assess the practical issues” | Hypertension, N=1 | Case study (n of 1 study) | | None | Nintendo Wii Fit, physical activity | - Mechanics: various games including bowling, tennis, baseball, golf, and boxing | Daily physical activity (sensor data from wearable device), exercise self-efficacy (self-report), motivation (self-report), quality of life (self-report), symptoms (heart faculty related, self-report) |
| Kurtzman et al [52] | “Our objective was to conduct a randomized, controlled trial to test the effectiveness of a gamification intervention that used insights from behavioral economics to enhance social incentives, such as collaboration, accountability, and peer support to promote weight loss” | Obesity, N=196 | Clinical trial with randomization into 3 groups: serious game, serious game plus sharing data with primary care provider, or control | | Behavioral economics | LOSE IT! weekly weight targets for individuals who were paired in teams of 2 | - Mechanics: weekly weight goals. - Action points: weekly weigh-ins with a wireless weight scale. - Feedback: points and leveling up | Weight loss (examination), physical activity (sensor data from wearable device) |
| Lyons et al [53] | “This study will test a conceptual model that consists of two complementary pathways: one involving narrative, identity, and persuasion, and another involving playfulness and intrinsic motivation” | Cancer, N=120, planned | Protocol for clinical trial randomized into 2 groups: serious game or active self-monitoring group | | Self-determination theory, narrative transportation theory | Zombies, Run! (off-the-shelf) encourages walking, jogging, or running | - Mechanics: user given missions to pick up supplies to avoid zombies. - Mood: user selects music. - Feedback: summaries after missions, rewards, and points | Physical activity (sensor data from wearable device and self-report), fitness (examination), exercise role identity (self-report), quality of life (self-report), mood (self-report), symptoms (fatigue, sleep; self-report), motivation (self-report) |
| Radhakrishnan et al [54] | “We present a Heart Health game prototype - an application for mobile tablets - and we assess the game’s usability and evaluate its functionality for improving heart failure self-management knowledge and behaviors among community-dwelling older adults with heart failure” | Hypertension, N=19 | One-group, pretest and posttest feasibility study | | Gagne’s instruction strategies | Heart health game | - Mechanics: heart failure self-management information presented within casino slot machines where points can be earned and used to bet. - Mood: 4 rooms to play slot machines. - Feedback: reward and incentive, leveling up | Self-management knowledge (self-report), heart failure self-management (self-report) |
| Senior et al [55] | “To investigate the effectiveness of participation in a Nintendo Wii tennis group activity on physical activity levels of sedentary people with type 2 diabetes mellitus. Secondary aims were changes in health status, quality of life, fitness, safety; and participants’ experience” | Diabetes, N=11 | One-group, pretest and posttest feasibility study | | None | Nintendo Wii Fit tennis game, exergaming | Game features assumed | Physical activity (self-report), BMI (examination), quality of life (self-report), symptoms (self-report) |
| Shin et al [56] | “The objective of the present study was to determine whether health-related quality of life depression, and upper extremity function could be improved using game-based virtual reality rehabilitation plus conventional occupational therapy, compared to the same amount of conventional occupational therapy alone” | Stroke, N=32 | Clinical trial with randomization into 2 groups: serious game or conventional occupational therapy | | None | RehabMaster, rehabilitation | - Mechanics: arm movements | Quality of life (self-report), mood (self-report) |
| Shin et al [57] | “The objective of the present study was to examine the effects of virtual reality-based rehabilitation combined with standard occupational therapy on distal upper extremity function and health-related quality of life, and compare the findings to those of amount-matched conventional rehabilitation in stroke survivors” | Stroke, N=46 | Clinical trial with randomization into 2 groups: serious game or occupational therapy | | None | RAPAEL Smart Glove: a biofeedback system designed for distal upper extremity rehabilitation in stroke survivors | - Mechanics: simulate activities of daily living (eg, squeezing oranges, cooking, cleaning the floor, and turning over pages). - Feedback: visual | Quality of life (self-report) |
| Simmons et al [58] | “To measure both motor recovery and cognitive enhancement of an adult population in the chronic stage of recovery from acquired brain injury” | Stroke, N=12 | One-group, pre- and posttest feasibility study | | Simulation theory | PreMotor exercise games | - Mechanics: actions that simulate real-life tasks such as opening a box, maneuvering a light bulb, jigsaw puzzle, and letter games | Cognitive function (examination) |
| Simsek et al [59] | “Our study was designed to investigate the influences of Nintendo Wii play systems targeting upper extremity function and balance and conventional therapy on dependence in daily living activities and health-related quality of life” | Stroke, N=42 | Clinical trial with randomization into 2 groups: serious game or conventional physiotherapy | | None | Nintendo Wii Fit, physical capacity | - Mechanics: 5 gamses selected from package to promote upper limb and balance training | Quality of life (self-report) |
| Song et al [60] | “This study aimed to determine the effects of training using virtual reality games on balance and gait ability, as well as the psychological characteristics of stroke patients, such as depression and interpersonal relationships, by comparing them with the effects of ergometer training” | Stroke, N=40 | Clinical trial with randomization into 2 groups: serious game or ergometer training group | | None | Xbox Kinect, exergaming | - Mechanics: various sports programs including bowling, skiing, walking, and climbing stairs | Mood (self-report), relationship changes (self-report) |
| Sutanto et al [61] | “The primary aim of this preliminary randomized controlled trial was to evaluate the clinical effects of the addition of interactive video games with a Wii Fit balance board as a part of an exercise training program” | COPD^d^, N=20 | Clinical trial randomized into 2 groups: serious game or hospital-based outpatient exercise training program | | None | Nintendo Wii Fit, exergaming | - Mechanics: 3 games for physical exercise (yoga, strength training, and aerobic exercise). - Feedback: confirm compliance, score to motivate, and evaluation on the screen | Exercise tolerance (examination), symptoms (self-report), quality of life (self-report), BMI (examination), airflow obstruction (examination), exercise capacity (examination), cost (medical record) |
| Towle et al [62] | “We report here on the features of games we felt were useful and on the disadvantages of using computer games with stroke patients” | Stroke, N=11 | One-group, pretest and posttest feasibility study | | None | Computer-based cognitive rehabilitation | - Mechanics: 16 games (visual or verbal) including memory of pictures, words, faces, word list recall, and memory of a map. - Feedback: musical noises for correct and incorrect choices | Cognitive function (multiple examinations) |
| van Balkom et al [63] | “The main research questions of this project are 1) What is the short-term and long-term effect of cognitive training on objective and subjective cognitive functioning in Parkinson's Disease? and 2) What are the neural mechanisms underlying the effect of cognitive training in Parkinson's Disease?” | Parkinson disease, N=140, planned | Protocol for clinical trial randomized into 2 groups: serious game or active control (3 games with no specific cognitive engagement) | | Cognitive training reorganization on brain network infrastructure | BrainGymmer web cognitive trainingplatform modified for study, train cognitive abilities (executive function, working memory, attention, and processing speed) | - Mechanics: 13 cognitive training games with dynamic difficulty adjustment. - Feedback: presented after the first 9 games are completed | Physical activity (self-report), medication adherence (medical record), cognitive function (multiple examinations), symptoms (self-report), mood (self-report) |
| van de Weijer et al [64] | “The aim of this study is to determine whether a web-based gaming service designed for cognitive training is a feasible approach, and able to improve cognitive functioning within a three-month time frame in a new cohort of Parkinson’s Disease-Mild Cognitive Impairment patients” | Parkinson disease, N=222, planned | Protocol for clinical trial randomized to serious game or wait list | | None | MyCognition AquaSnap | - Mechanics: taking pictures of ocean animals, having missions to discover new areas of the ocean. - Feedback: pictures users take are worth currency that can be used to dive deeper into the water. - Feedback: see progression on map, coins | Cognitive function (multiple examinations), mood, symptoms (multiple self-report surveys), quality of life (self-report) |
| van Santen et al [65] | “The aim of our study is to investigate the effectiveness and cost-effectiveness of exergaming compared to regular activities in people living with dementia, who attend day-care centres. Additionally, we want to investigate whether the exergaming activity for the person living with dementia, also (indirectly) affects the informal caregiver” | Dementia, N=224 dyads (patients with dementia and informal caregiver), planned | Protocol for clinical trial randomized into 2 groups: serious game or regular activities at day care centers without exergaming | | None | To be determined off-the-shelf game, but will include cycling, exergaming | - Mechanics: cycling in familiar cities. - Mood: enjoyable and relaxing scenes | Physical activity (self-report), mobility (examination), cognitive function (examination), social function (examination, self-report), motivation (self-report), BMI (examination), fall incident rate (medical record), costs (medical record, self-report), caregiver outcomes similar to those of patient (self-report, examination) |
| Wang et al [66] | “The aims of the study included the following: Aim 1: To assess the feasibility, acceptability, and safety of the PAfitME intervention in head and neck cancer patients after cancer treatment.  Aim 2: To describe adherence rates during the 6-week PAfitME intervention in head and neck cancer patients after cancer treatment.  Aim 3: To analyze changes in cancer-related fatigue, activities of daily knife dependence, and fitness performance at 6 weeks” | Cancer, N=8 | One-group, pretest and posttest feasibility study | | Intervention mapping approach | PAfitME, physical activity | Game features assumed | Symptoms (fatigue, self-report); adherence (data from game system), cardiorespiratory fitness (examination) |
| Yu et al [67] | “The purpose of this experimental study was to evaluate the preliminary efficacy of Memory Matters that was delivered one on one versus in a group format on mood, social interaction, quality of life, and behavioral and psychological symptoms in dementia in people with dementia” | Dementia, N=80 | Clinical trial randomized into 3 groups: wait-list control, serious game with groups of 2-3 participants, serious game one-on-one with interventionist | | None | Memory Matters, reminiscence game to interactive activities designed to tap long-term memories | - Mechanics: matching game with familiar images, sounds, and music; can play solo or with others; and can use personal photos. - Mood: familiar objects tailored to age group. - Action points: matching game or slide show. - Feedback: correct matches recognized | Mood (self-report), quality of life (self-report), social interaction (self-report), symptoms (behavioral and psychological symptoms in dementia, self-report) |

^a^These 2 articles by da Silva Alves et al [33,34] report findings from the same sample (n=45) in the same study.

^b^HbA_1c_: hemoglobin A_1c_.

^c^These 2 articles by Hӧchsmann et al [41,42] report the different outcomes of a single study. The protocol for this study was also published but was not included in this review.

^d^COPD: chronic obstructive pulmonary disease.
